# Emerging Encapsulation Technologies for Long-Term Reliability of Microfabricated Implantable Devices

**DOI:** 10.3390/mi10080508

**Published:** 2019-07-31

**Authors:** Seung-Hee Ahn, Joonsoo Jeong, Sung June Kim

**Affiliations:** 1 Department of Electrical and Computer Engineering, Seoul National University, Seoul 08826, Korea; 2 Department of Biomedical Engineering, School of Medicine, Pusan National University, Yangsan 50612, Korea; 3 Institute of Aging, College of Medicine, Seoul National University, Seoul 08826, Korea

**Keywords:** biomedical implants, encapsulation, packaging, micro package, long-term reliability

## Abstract

The development of reliable long-term encapsulation technologies for implantable biomedical devices is of paramount importance for the safe and stable operation of implants in the body over a period of several decades. Conventional technologies based on titanium or ceramic packaging, however, are not suitable for encapsulating microfabricated devices due to their limited scalability, incompatibility with microfabrication processes, and difficulties with miniaturization. A variety of emerging materials have been proposed for encapsulation of microfabricated implants, including thin-film inorganic coatings of Al_2_O_3_, HfO_2_, SiO_2_, SiC, and diamond, as well as organic polymers of polyimide, parylene, liquid crystal polymer, silicone elastomer, SU-8, and cyclic olefin copolymer. While none of these materials have yet been proven to be as hermetic as conventional metal packages nor widely used in regulatory approved devices for chronic implantation, a number of studies have demonstrated promising outcomes on their long-term encapsulation performance through a multitude of fabrication and testing methodologies. The present review article aims to provide a comprehensive, up-to-date overview of the long-term encapsulation performance of these emerging materials with a specific focus on publications that have quantitatively estimated the lifetime of encapsulation technologies in aqueous environments.

## 1. Introduction

For decades, the field of implantable biomedical devices has been rapidly advancing, resulting in improvements to the diagnostic and therapeutic capabilities of a wide range of technologies including cardiac pacemakers, cochlear implants, deep brain stimulations, neural prostheses, and various physiological sensors and monitors [1,2,3,4,5,6,7,8,9,10,11,12]. The application of microfabrication technologies to medical implants has led to the development of devices with micro- or nanoscale precision and significantly reduced footprints and power requirements, but also increased capability and modality. One of the major technical challenges facing such miniaturized implants is long-term reliable packaging or encapsulation technology, given they are expected to remain stable, safe, and functional over a certain lifetime, which usually spans multiple decades.

To date, medically approved implantable devices have relied on titanium or ceramic cases for the hermetic encapsulation of active electronics. However, these packages are bulky, heavy, labor-intensive to produce, and difficult to miniaturize, making them unsuitable for microfabricated devices. The need for feedthroughs connecting the inside and outside parts of conventional metal or ceramic packages is another limitation that hinders scaling up toward large-count, high-density interfaces with biological media for sensing and stimulation.

A variety of organic and inorganic materials and related technologies have been proposed as alternative encapsulation methods for miniaturized biomedical implants as they exhibit mechanical flexibility, compatibility with the microelectromechanical system (MEMS) process or batch process, good electrical insulation, and conformal encapsulation of complex topography on top of the basic requirements of biocompatibility and long-term stability in vivo. Additionally, these thin-film coatings add minimized volume and weight to the devices. These emerging materials include inorganic thin-film coatings of Al_2_O_3_, HfO_2_, SiO_2_, silicon carbide (SiC), and diamond, as well as organic polymers such as polyimide, Parylene, liquid crystal polymer (LCP), silicone elastomer, SU-8, and cyclic olefin copolymer (COC). Methods of deposition of these materials are diverse, ranging from spin coating, chemical vapor deposition, casting, thermal lamination, and thermal growth to atomic layer deposition (ALD).

Although none of these materials have yet been proven to be as hermetic as metal packaging nor widely utilized in regulatory approved devices for chronic implantation, a number of studies have endeavored to investigate the feasibility of their use and have demonstrated promising outcomes in terms of their long-term reliability through a multitude of testing methodologies with great potential for further improved longevity.

Despite these encouraging results, to the best of our knowledge there are no review articles specifically focusing on the encapsulation performance of these emerging encapsulation materials. While many review articles have introduced and compared a series of biocompatible materials that are commonly used for implantable devices, their scope is relatively broad; for the most part, the general properties of the materials are discussed [13,14,15,16,17,18,19]. The present review, therefore, aims to provide a comprehensive overview of the various emerging packaging materials with a specific focus on their encapsulation performance. For this purpose, we primarily selected and compared publications that have not only demonstrated device encapsulation but also investigated long-term reliability in aqueous environments to provide a quantitative estimation of the expected lifetime of the encapsulation through accelerated aging or other scientific procedures.

## 2. Long-Term Reliability Test Principles

The long-term reliability of encapsulation materials is generally evaluated in accelerated aging experiments by soaking test samples in saline solution of elevated temperatures. The lifespan of the encapsulation, or the mean time to failure (MTTF), during accelerated aging can be translated into the equivalent lifetime at body temperature using the Arrhenius reaction rate function, which describes the temperature dependence of the chemical reaction rate, *k*, as follows:

$$k={\mathrm{Ae}}^{-E_{a}/RT},$$

where A is a constant, E_a_ is the activation energy, *R* is the gas constant, and *T* is the temperature in Kelvin. Therefore, the accelerating factor, *k*_1_/*k*_2_, of the raised test temperature, *T*_1_, with respect to body temperature, *T*_2_, can be obtained as follows:

$$\frac{k_{1}}{k_{2}}=\exp\left[\frac{E_{a}}{R}\left(\frac{1}{T_{2}}-\frac{1}{T_{1}}\right)\right].$$

The activation energy can be determined if the reaction rates (or MTTF values) at two different test temperatures are provided. As an approximation of the Arrhenius relationship for polymer reactions, the “10-degree rule” is commonly adopted to extrapolate the lifetime of polymeric materials at 37 °C. This rule states that the chemical reaction rate will double for every 10-degree increase in temperature, assuming the aging process is first-order or pseudo–first-order [20,21]:

$$\frac{k_{1}}{k_{2}}=2^{\left(T_{1}-T_{2}\right)/10\;}.$$

It is recommended that the accelerating temperature is kept below 60 °C because the accuracy of the 10-degree rule decreases with greater deviation from the ambient temperature. In addition, higher temperatures can introduce new failure mechanisms that would not occur at the normal operation temperature of 37 °C [20,21]. However, the 10-degree rule is still useful to predict the worst-case lifetime, given that it is known to give a conservative estimation over a wide range of temperatures specifically for most polymeric materials [20,22].

Common metrics for quantifying encapsulation include leakage current, electrochemical impedance spectroscopy (EIS), and the functionality of working devices. Leakage current measured between a pair of encapsulated interdigitated electrodes (IDEs) under DC bias is the most sensitive measure of moisture penetration. An initial current in the typical range of a few to tens of pA abruptly soars into the nA or μA range as a result of failed encapsulation. Additionally, EIS data, which is also usually measured between IDEs under encapsulation, can provide more comprehensive information about the degradation of the encapsulation when the EIS values are fitted into an equivalent circuit model of the encapsulation. Changes in each circuit component can be used to investigate the failure mechanisms of, for example, dissolution, blistering, ion transport, or pore formation [23,24]. Functional devices such as recording or stimulating electrode arrays [25,26], neural recording systems with a wired or wireless connection, or wirelessly interrogatable tiny chiplets [27,28,29] can also be used as test vehicles in encapsulation assessment. While these functional devices can provide more practical and exact predictions of the lifetime of certain applications than specially designed test samples, this method is not suitable for investigation of encapsulation performance itself because the loss of the functionality of the full system is associated with various factors other than failed encapsulation.

Lastly, presence of electrical bias generated by wired or wireless powering can introduce another mode of stress accelerating the failure process of the encapsulation. Electrical stress caused by voltage gradient or current flow can facilitate the electrochemical process including corrosion, material degradation, ion movement, and water electrolysis [30,31]. While the effect of electrical bias on the encapsulation performance has not been extensively investigated yet, this is an essential part in the field as most of the active implantable devices need electrical powering.

## 3. Inorganic Materials

A summary of MTTF values from long-term reliability tests of encapsulation with various inorganic materials is presented in Table 1.

### 3.1. Al_2_O_3_

Aluminum oxide (Al_2_O_3_), known as alumina, is a well-proven biocompatible material that has long been used for packaging of implantable devices, typically in the encasement of electronics or as a feedthrough plate with a brazed titanium package. Despite its good properties as a barrier and the RF transparency of ceramic packaging, its application in microfabricated devices is limited due to difficulties in miniaturization and the necessity of a labor-intensive manual assembly process. Instead, thin films of Al_2_O_3_ created by atomic layer deposition (ALD) technology typically at 120 °C [26,28] have recently gained attention as a way to overcome the restrictions of bulk alumina. This method provides not only excellent moisture barrier properties and nanoscale precision but also highly conformal coverage of irregular surfaces and pinhole-free film quality. This has made Al_2_O_3_ an attractive encapsulation material for moisture-sensitive organic light emitting diodes. Recently, ALD Al_2_O_3_ has been explored as a moisture barrier material for implantable microdevices. Since Al_2_O_3_ is known to suffer from hydrolysis in direct contact with liquid water [39], a combination of ALD Al_2_O_3_ and organic polymers has been proposed. In this configuration, Al_2_O_3_ layers act as an inner moisture barrier and polymer layers, e.g., polyimide or Parylene C, act as an external barrier, preventing exposure of the Al_2_O_3_ to water.

In some studies, accelerated aging tests have been conducted to demonstrate the enhanced lifetime of the Al_2_O_3_/polymer bilayer scheme compared to conventional polymer-only barriers [23,28,30,32]. Xie et al. has pioneered the research in this area by performing a set of comprehensive studies using various types of testing vehicles (IDE, UEA, and wirelessly functional neural interfaces) and various evaluation criteria (EIS, leakage current, and RF signal) at multiple aging temperatures [27,28,30,32]. With an IDE structure, a 52 nm-thick layer of ALD Al_2_O_3_ improved the MTTF to 180 days in 80 °C saline, compared to 35 days with a 6 μm-thick Parylene C coating [30,32]. A similar test using a wirelessly functional neural interface suggested that the bilayer structure enhanced the lifetime tenfold compared to a Parylene-only coating either with or without continuous 5 V powering [28]. It was also suggested that an alumina coating thicker than 32 nm did not make a noticeable difference in barrier performance [30]. Minnikanti et al. also reported that the lifetime of an ALD (52 nm)–Parylene (6 μm) bilayer, 215 days in 60 °C saline, was five times greater than that of a Parylene-only coating [23]. Test samples with various topographical features such as tines, partial silicone potting, and openings over IDE chips or UEA were created and coated with Parylene only or with an Al_2_O_3_ + Parylene bilayer. The bilayer structure extended the MTTF by a factor of three to five depending on topography at the aging temperature of 57–67 °C [26].

### 3.2. HfO_2_

An HfO_2_ coating is usually formed by the ALD process which can provide a conformal coating over nanoscale structures with pinhole-free and atomically dense film quality. ALD-deposited HfO_2_ layers are known to be chemically, thermally, and mechanically stable, optically transparent from 300 to 10,000 nm, and with a tunable refractive index [40,41,42,43]. A high dielectric constant of approximately 20 [44] has been noted by the semiconductor industry to utilize HfO_2_ as a high-κ gate dielectric material to replace SiO_2_ in ultra-small complementary metal-oxide-semiconductor technology [40,41,45]. Use as an optical coating material is another major application of HfO_2_ [43,46]. With positive results regarding the in vitro biocompatibility of HfO_2_ in terms of cytotoxicity, hemolysis, and cell imaging [47,48,49] along with the inherently excellent property of HfO_2_ as a barrier against liquid water, its applications have been extended to include uses in biological devices, such as in biocompatible passivation or the functionalization of biosensors [49,50,51,52].

Recently, HfO_2_ has been demonstrated to be a hermetic packaging material for microfabricated implantable devices that either utilize HfO_2_ as an additional moisture barrier between organic materials like polyimides [33,34] or employ a homogeneous ALD HfO_2_ or HfO_2_/SiO_2_ multistack as an encapsulation layer [29]. The addition of two 8 nm-thick ALD HfO_2_ layers between polyimide (PI) and ALD AL_2_O_3_ layers (i.e., PI/HfO_2_/Al_2_O_3_/HfO_2_/PI) improved the water vapor transmission rate (WVTR) of the polyimide-only films from 4300 mg/cm^2^∙day to a value below the detection limit of the WVTR equipment (0.5 mg/cm^2^∙day), determined by the Mocon method [33,34]. The WVTR of the film with a HfO_2_ barrier was also lower than that of PI layers with a single Al_2_O_3_ layer (4 mg/cm^2^∙day) or two Al_2_O_3_ layers (1.7 mg/cm^2^∙day). These results, despite being preliminary, suggest the potential usefulness of ALD HfO_2_ layers as a moisture barrier for implantable devices.

More recently, alternating layers of 10 nm-thick HfO_2_ and SiO_2_ up to 100 nm in total thickness, deposited at 250 °C, have been demonstrated to provide conformal and stable encapsulation of sub-millimeter-sized wirelessly functional implantable silicon chiplets (ASICs) [29]. This approach has the potential for great scalability; hundreds of chiplets could be suspended in wireframe mesh for simultaneous conformal coating of hundreds of devices. The long-term encapsulation performance of this ALD coating was evaluated in an accelerated aging test in which the ALD-coated active devices were soaked in 87 °C saline while wirelessly interrogated to monitor any functional degradation as a result of moisture infiltration through the ALD coating. The chiplets failed to function after 185 days on average, presumably due to pinholes or nano cracks rather than dissolution of the HfO_2_ layer. ALD HfO_2_/SiO_2_ encapsulation with opening windows over two electrode pads survived for more than 100 days and were still ongoing in 87 °C saline.

### 3.3. SiO_2_

Packaging of implantable electronics within tubes, beads, or lids made from shaped glass or fused silica has been widely used with the typical bonding methods of laser welding and anodic bonding [35,36,37,53,54,55,56,57]. Despite the advantages of inertness, low permeability, well-established bonding techniques, and RF and optical transparency, this macroscale glass packaging is bulky, heavy, and incompatible with the microfabrication process, thus limiting its application in miniaturized implantable devices.

Thin film SiO_2_ layers of a few hundred nanometers deposited by semiconductor technologies including chemical vapor deposition (CVD), ALD, and thermal oxidation of silicon have recently been proposed as a potentially suitable candidate for the encapsulation of implantable electronics. SiO_2_ is known to slowly dissolve by hydrolysis in salty water like bodily fluids, albeit with greatly varying dissolution rates depending on the deposition method. A recent study demonstrated that SiO_2_ layers thermally grown on the Si wafer could offer outstanding barrier properties compared to SiO_2_ layers formed by plasma-enhanced CVD (PECVD) or e-beam evaporation. Accelerated aging of the thermally grown SiO_2_ at multiple temperatures (~1100 °C) showed dissolution rates of 80 nm/day at 90 °C and 5.6 nm/day at 70 °C, which can be extrapolated to a few decades of lifetime at body temperature with a 1 μm-thick film [35]. The SiO_2_ layer was transferred onto a flexible electronics platform such that resistors, capacitors, diodes, and metal-oxide-semiconductor transistors could be encapsulated before immersion in 90 °C PBS. These devices failed after 12 days, consistent with the above dissolution rate of SiO_2_ layers [35,55]. Another study reported a very slow dissolution rate of 0.104 nm/day at 87 °C saline of a thermal SiO_2_ coating protecting a photovoltaic retinal implant [36]. Thermal SiO_2_ has been commonly shown to be much more stable in saline than low-pressure CVD (LPCVD) SiO_2_, which was dissolved at the rate of 1 nm/h at 90 °C [37]. Additionally, thin layers of SiO_2_ have been proposed to form an enhanced moisture barrier in combination with other inorganic materials including HfO_2_, silicon nitride, and SiC [29,37,55].

### 3.4. SiC

Silicon carbide (SiC), specifically amorphous SiC (a-SiC), is typically deposited by sputtering or PECVD up to thicknesses of a few micrometers. SiC has good barrier properties against diffusion and dissolution in saline, a low deposition temperature, good physical robustness, chemical inertness and thermal stability, high flexibility in thin-film form, and compatibility with MEMS processes [58,59,60]. The biocompatibility of SiC has been demonstrated to be much better than that of silicon and as good as that of titanium [61,62]. Amorphous SiC has recently been tested in various neural prosthetic applications not only as a conformal encapsulation material [36,58,63] but also as adhesion-promoting layer of a polymer coating [63,64] and as both substrate and an insulating material of neural interfaces, enabling all-SiC devices [65,66]. Additionally, a-SiC has been used in a recently regulatory approved cardiac stent [67].

Some of the aforementioned studies involved long-term stability assessments. SiC films 67 nm thick and deposited by PECVD typically at 200 to 400 °C were soaked in 90 °C PBS and measured over time to estimate the dissolution rate. The SiC films showed a dissolution rate of 0.1 nm/h, which was tenfold less than that of SiO_2_ (1 nm/h) and Si_3_N_4_ (2 nm/h) under the same aging conditions. Aging at 37 °C did not result in any thickness reduction of the SiC film over 40 weeks of soaking [37]. Another study using a photodiode electrode array encapsulated by 1.4 μm-thick SiC film also found that PECVD a-SiC films showed no sign of dissolution over 16 weeks of accelerated aging at 87 °C [36]. Silicon-based UEA encapsulated by SiC film 550–750 nm thick survived more than 6 weeks at 90 °C [38].

### 3.5. Diamond

Diamond has been considered as an alternative biomaterial due to its mechanical hardness, wear resistance, and chemical resistance [68]. Polycrystalline diamond (PCD) is the most commonly used form of diamond in the industry, typically fabricated by sintering diamond grit under high pressure and temperature. For packaging of implantable electronics, PCD plates are processed by laser milling to create the substrate, caps, or feedthrough that are bonded by laser welding or brazing [69,70,71,72]. Ultrananocrystalline diamond (UNCD) is another type of diamond which can be deposited by PECVD at 400 °C on which a conductive area can be formed by N-doping [69,70,73,74,75]. A package with feedthrough made from a combination of PCD and UNCD has been tested for encapsulating retinal prostheses [69,70]. While the long-term reliability of diamond-based packaging has yet to be tested, some groups have evaluated its biocompatibility and chronic tissue response through an in vitro cytotoxicity test and in vivo biocompatibility tests. The in vivo test using UNCD-coated silicon chips implanted in the eyes of rabbits for 6 months [73] and the abdominal cavities of mice for 3 months [74] have proven the bioinertness and biostability of the UNCD coating.

Although it is not a diamond, diamond-like carbon shares some of the typical properties of diamond and has also been suggested as an encapsulation material for implantable devices [76,77,78].

## 4. Organic Materials

The representative material properties of organic materials introduced in this article are compared in Table 2, and a summary of the MTTF values from long-term reliability tests of encapsulation with various organic materials is presented in Table 3.

### 4.1. Polyimide

Polyimide is a branch of commercially available polymers and is literally a polymer of imide monomers that is available in the form of film, tape, or spinnable liquid. Polyimide features excellent thermal and chemical stability, high glass transition temperature, and flexibility. Among the several types of polyimide, depending on the type which vary according to their building blocks (dianhydride, diamine, etc.), the BPDA/PPD type is the most commonly used for medical applications. Although it is not certified according to the ISO 10993, various groups have proven its biocompatibility and low cytotoxicity [15,93,94,95], placing the polyimide among the most widely used substrate and encapsulating polymer for neural interfaces [96,97,98,99,100,101].

Encapsulation using polyimide is based on spin coating on the substrate followed by curing at ~400 °C. Due to its excellent thermal and chemical stability, polyimide is compatible with most of the MEMS batch process. Photolithography using photosensitive polyimide [102,103,104], liftoff using sacrificial layer [101], and bonding using polyimide as an adhesion [105,106,107] are available techniques for polyimide.

Some of those studies have quantitatively evaluated the long-term reliability of polyimide encapsulation under accelerated aging conditions. Test samples with IDE patterns sandwiched between 10 μm-thick polyimide layers were soaked in 75 °C phosphate buffered solution (PBS) saline for accelerated aging, while the leakage currents between IDE channels were measured to detect any encapsulation failure. The leakage current exceeded the threshold of 1 μA after 66 days of soaking [85], which is roughly equivalent to a lifetime of 2.5 years at 37 °C based on the 10-degree rule. The failure modes were dissolution, delamination, blistering, and corrosion. In another study, test samples made of three different commercial polyimide products were soaked in PBS at 37, 60, and 85 °C, and in deionized water at 85 °C [86]. The mechanical properties of the samples, including Young’s modulus, fracture energy, stress at break, strain at break, and stress at 10% strain, were measured for more than 20 months. Over the study period, all the samples were stable in PBS at 37 and 60 °C without showing any changes in their properties relative to the control dry samples. On the other hand, degradation was observed in the samples in PBS at 85 °C, including mass loss and decreased mechanical properties [86]. In other studies, 64-channel micro-electrocorticographic (μECoG) electrode arrays fabricated by 5–12.5 μm-thick polyimide layers were soaked in 60 °C saline for an accelerated aging test [87,88]. With the failure criteria being the time point at which the number of working channels dropped below 50% of the initial working channels, two of three samples survived for over 300 days, resulting in a predicted lifetime of four to seven years [87].

### 4.2. Parylene

Parylene refers to a class of semi-crystalline polymers discovered in the 1940s and commercialized by the Gorham process about 20 years later which enabled room temperature deposition. Parylene can be deposited as a thin, conformal, pinhole-free film exhibiting flexibility, optical transparency, chemical inertness, and low water absorption (<0.1%). Among the commonly available types of parylene, parylene-N, parylene-C, and parylene-HT have acquired the ISO 10993, USP Class VI rating. Parylene-C is the most popular type owing to its lower moisture and gas permeability compared to Parylene-N [15,108,109], and has been used in a wide range of biomedical applications such as bladder volume sensors [110], microelectrode arrays [111,112,113], orthopedic implants [114,115], and dental implants [116,117].

Encapsulation using parylene is based on the CVD process. Due to the molecular level deposition process, a uniform and conformal film can be formed over complex sample surface topography, including sharp edges and crevices [118,119,120]. Parylene film is typically no thicker than 100 μm, which adds minimal volume and weight to the devices, but at the same time it cannot provide mechanical strength or robustness [15]. Therefore, parylene is sometimes used in combination with other encapsulation materials such as silicone elastomer [90], Al_2_O_3_ [26,30], and glass [89] to complement its mechanical properties.

Some studies have evaluated the long-term reliability of the parylene encapsulation under accelerated aging conditions. IDE test samples encapsulated by 10 μm-thick parylene-C layers soaked in 75 °C PBS failed after 117 days when the leakage current soared beyond the 1 μA threshold as a result of blistering and delamination [85]. According to the 10-degree rule, the expected lifetime of this sample device at 37 °C is about 4.5 years. In another study, test samples coated by parylene-C over a glass substrate were soaked in 85 and 97 °C saline for accelerated aging while the line resistance between multiple channels was measured. The samples failed after 31 and 15 days, respectively, with the threshold being a 50% change in the resistance value, which is equivalent to approximately 2.5 years at body temperature based on the 10-degree rule. The failure mode of the samples was moisture diffusion through the parylene barrier layers and undercut of the glass [89]. In another study, IDE test samples coated with 6 μm-thick parylene-C were soaked in 60 °C PBS with periodic electrochemical characterizations such as EIS, leakage current, and cyclic voltammetry every 6 h. The MTTF of the six samples was 1117 h, or 49.1 days, when the leakage current exceeded 1 nA [23]. Similar studies using IDE samples coated with 6 μm-thick parylene-C layers have reported an MTTF of 150 days in 57 °C, which is equivalent to 1.64 years at 37 °C [30], 49.1 days at 60 °C, or 0.66 years at 37 °C [23], and 110 days at 67 °C, or 2.41 years at 37 °C [26].

### 4.3. Silicone Elastomer

Silicone elastomer is a biostable synthetic polymer which has a backbone made of repeating silicon-oxygen bonds and methyl groups. Among a variety of silicone elastomeric materials, polydimethylsiloxane (PDMS) is the most commonly used silicone in micro- and nanoscale soft lithography including molding, contact printing, and imprinting in biomedical applications because of its advantageous properties such as high elasticity, optical transparency, adjustable surface composition, and biocompatibility. Dipping, casting, and molding are the most common techniques for silicone encapsulation which are followed by curing at typically from room temperature to ~150 °C.

High permeability to gases and vapors, although useful for some applications, may limit the application of silicone elastomer for encapsulation purposes. Nevertheless, the packaging performance of silicone elastomer can be further enhanced when it is used in conjunction with other materials such as glass, parylene, and metal [28,89,121]. Additionally, it can be used to make transparent, flexible, and stretchable bioelectrodes [90,122,123,124].

An additional 5 mm-thick coating silicone elastomer on top of a 40 μm-thick parylene-C layer could extend the expected lifetime of the test samples from 2.5 to 6.7 years at body temperature based on accelerated aging at 85 and 97 °C PBS, during which failure was defined as the resistance value falling below half of the initial value [89]. Recently, PDMS with its surface pores filled by parylene, so called “parylene-caulked PDMS,” has been shown to effectively suppress water permeation through PDMS [90]. When identical electrodes samples were soaked in 36.5 °C PBS for 209 days, impedance of the electrode samples coated with parylene-caulked PDMS remained within 20% of the initial values, while the impedance values of all samples coated in PDMS decreased almost to zero [90]. Despite the inadequate barrier properties of silicone elastomer, it could be useful as a secondary coating material given its good biochemical stability and mechanical properties.

### 4.4. LCP

LCP has increasingly gained attention as an emerging biocompatible material for substrate and packaging of implantable neural devices, primarily owing to its lower moisture absorption rate (<0.04%) than conventional biocompatible polymers such as polyimide (∼2.8%), parylene-C (0.06–0.6%), and silicone elastomers [91]. This advantageous property is expected to contribute to improving the long-term reliability of polymer-based biomedical implants when properly processed. The thermoplasticity of LCP can be utilized to create a non-planar structure for conformation to target tissues by a simple thermoforming process, and to rapidly form a multilayered structure by stacking independently prepared LCP layers and thermally pressing them to bond together. The potential of LCP has been demonstrated in wide range of applications in neural engineering such as a miniaturized all-LCP retinal implant with an eye-conformable structure [125] as well as various shapes of neural electrode arrays for cortical [126,127], cochlear [128,129], intraocular [130,131,132], and peripheral applications [133,134,135].

LCP encapsulation basically begins from LCP films, which are commercially supplied in rolled sheets of varying thicknesses but can be applied to both planar and non-planar packaging. For encapsulation of planar structures such as neural electrode arrays of LCP/metal/LCP configuration, a substrate LCP film of higher melting temperature (~335 °C) with metallized pattern is thermally bonded to a cover LCP layer of a lower melting temperature (282 °C) by heating and pressing them together at a temperature between their melting points. Multiple layers can be fabricated using lower-melting-temperature LCP film as adhesive layers. For non-planar shape such as for packaging of electronics assembled on the substrate LCP layer, the first LCP layer is thermally deformed into, typically, a domed shape, which is thermally bonded to the substrate LCP film by selectively applying heat pressure onto the perimeter of the package to create a conformal encapsulation [85,125]. Another approach has also been proposed: filling the cavity between the substrate and the lid with milled LCP powder followed by pressing of the entire area, which could avoid the complex tooling of selective pressing [85].

Some studies have evaluated the long-term reliability of the LCP encapsulation in accelerated aging conditions. Test samples with IDE patterns of the basic LCP/metal/LCP sandwiched structure were soaked in 75 °C saline for accelerated aging while the leakage current was measured to detect any moisture penetration. The samples were failed after 379 days in 75 °C saline when the leakage drastically soared up above the 1 μA threshold, presumably due to water infiltration through the LCP-LCP bonding which resulted in complete delamination of two LCP layers as observed in the failed samples [25,85]. Test samples having opening windows like neural electrodes were also subjected to the aging condition in 87 °C saline by observing the voltage transient while stimulation pulses were continuously applied, which failed after 114 days on average as confirmed by the loss of the voltage waveform as a result of water penetration and electrically shorting of two channels. Non-planar package samples with IDE pattern, mimicking the circular dome-shaped package part of the all-LCP retinal implant, failed after 87 days in 87 °C saline. These results imply that the weakest interface against water penetration for this type of polymer encapsulation is polymer-metal adhesion around the channel openings rather than water ingression via the polymer-polymer adhesion and permeation through the bulk polymer surface. Microscale interlocking structure on the gold sites around the openings was reported to improve the MTTF of the device from 185 to 224 days at 75 °C saline, by providing mechanical interlocking between LCP and metal to enhance the bonding strength between them and thus be more resistant to water infiltration [92].

### 4.5. SU-8

SU-8 is a photoresist based on epoxy resin including a photo-acid generator compound and incorporated solvent which was first developed as a thick photoresist for microelectronics to achieve a high aspect ratio. It is transparent for visible light and is chemically and mechanically stable. Several studies using the baseline battery of ISO 10993 found that implantation of SU-8 pieces in vivo caused minimal histological irritations to surrounding tissues [13]. These results, however, have not strictly satisfied all the required specifications to meet the full ISO 10993 standard [13], and SU-8 has not yet attained the USP Class VI rating for biocompatibility. Despite a cytotoxicity problem related to antimony-based leachates originating from its chemical composition, some studies have reported that the leaching amount is below the safety limits recommended by the United States Environmental Protection Agency (USEPA) [136]. Nevertheless, due to its mechanical, chemical stability, and MEMS process compatibility, SU-8 has been diversely utilized especially as a high aspect-ratio structuring material instead of silicon in biomedical applications [15]. The mechanical strength, optical transparency, ease of patterning with MEMS compatibility also promoted the use of SU-8 for the substrate and encapsulating material for biomedical devices such as microelectrode arrays [13,137,138,139,140], optrode [141,142,143], and other implantable devices [144,145]. Encapsulation using SU-8 is typically based on spin coating followed by photolithographic patterning. [146]. While a long-term reliability evaluation of SU-8 based encapsulation has yet to be published, SU-8 has a potential as a good alternative for the organic packaging technologies considering its chemical inertness.

### 4.6. COC

COC is a kind of engineering thermoplastic with very low moisture absorption rate (<0.01%), which is lower than LCP. An in vitro biocompatibility test has shown the cytotoxicity of COC meets ISO 10993 requirements, suggesting the COC as a new candidate material for medical applications [147].

Optical transmittance of COC higher than 92% in the wavelength range of 300–1100 nm [148] has first encouraged various biomedical studies to explore the COC in optical applications such as optical fiber for biosensing or optrode for implantable optogenetic devices [149,150,151,152]. The first use of COC for encapsulation as well as substrate material of implantable device has demonstrated a COC-based multichannel neural probe in which a cover COC film is thermally laminated onto the substrate COC film with gold patterns at 235 °C. [148]. Although no long-term reliability test has been reported as far as we understand, the exceptionally low moisture absorption rate of COC is certainly the most promising property of COC as an emerging packaging material that can provide both good hermeticity and optical transparency.

## 5. Conclusions and Outlook

A summarized overview on the recent status of the long-term encapsulation performance of the new kinds of organic (parylene, polyimide, LCP, silicone elastomer, COC) and inorganic (Al_2_O_3_, HfO_2_, SiO_2_, SiC, and diamond) biocompatible materials was presented with a specific attention on the quantitatively assessed reliability in an aqueous environment. Compared to conventional macroscale packages using metal or ceramic, those emerging materials can provide advantages such as compatibility with miniaturized devices typically through MEMS processes, manufacturability, or conformal encapsulation over microscopic surface topography with minimized addition of volume. Although extensive efforts have recently developed various encapsulation technologies using new or existing materials to demonstrate fairly promising estimation of the long-term reliability of the encapsulation through accelerated aging conditions, many challenges still remain in terms of both the material properties and processing technologies. While each material and processing scheme has its own pros and cons, for example, none of the devices with the new materials have been chronically implanted in a physiological environment in a large number of patients so far. A few examples of these materials having been used for patients in clinical trials of active implantable devices include parylene for Utah probes [153,154], Argus II retinal implant [155], and polyimide for epiretinal [156] and subretinal implant [157,158], which have been tested, at most, for a few years. Additionally, the accelerated aging procedure in hot PBS commonly adopted in the studies discussed in this article may not be able to account for the hydrolytic, oxidative, and enzymatic degradation of those materials, especially organic materials, in the harsh environment of the actual implanted condition. Recently, the reactive accelerated aging (RAA) method has been introduced to mimic the presence of the reactive oxygen species (ROS) in the implanted physiological condition by adding hydrogen peroxide in PBS of the conventional accelerated aging tests [159,160].

Sterilization would be another issue for the clinical application of those new materials. The organic materials are thought to be more susceptible to the sterilization process than the inorganic materials. As many of the materials mentioned above are at the research phase, however, investigations on the sterilization of those materials have not yet been extensively conducted. Stability of polyimide against the sterilization process has been tested to show that thermal or thermos-oxidative sterilization methods have induced degraded stability of polyimide, whereas the radiative method has contributed to generation of additional cross-linking and thus better stability under certain conditions [161,162]. When common sterilization processes have been tested on paryelene-C, e-beam induced breakage of chemical bonds, ethylene oxide (EO) changed the surface property, and autoclave affected polymer crystallinity [163]. In another study, autoclaving induced delamination, gamma sterilization changed chemical properties, and EO gas treatment generated clefts in the surface [164]. Nevertheless, it is still hard to correlate how the sterilization process affects the long-term reliability of the device. While reasonable compatibility of other inorganic and inorganic materials is expected, given the temperature stability of those materials at 200 °C at least, further studies are required to systematically investigate how the sterilization procedures affect the material integrity and long-term reliability.

However, despite the limitations of the emerging encapsulation materials and technologies to date, we believe the outlook of those new materials for replacement of the conventional packaging technologies for chronically implantable biomedical devices is still promising. This is because the properties and barrier performance of the encapsulation created by those emerging materials can be widely tunable, adjustable, and engineered for optimized characteristics. Continued multidisciplinary studies are expected to address the existing problems that have been revealed so far and also to introduce completely novel materials and creative processes that will overcome the current limitations in the field of implantable biomedical devices. One of those efforts would be multi-combinational studies of various organic and inorganic materials. Multilayered organic (e.g., parylene + elastomers) or inorganic (e.g., HfO_2_ + SiO_2_) coatings have been introduced in this paper, but a combination of multiple organic and inorganic materials will offer a greater potential towards the hermetic encapsulation by higher degree of tunability on the material characteristics. While combinational organic and inorganic coatings of Al_2_O_3_ + parylene and HfO_2_ + polyimide have been introduced, one drawback of those approaches is that the organic and the inorganic layers are deposited in different chambers, for example, an inorganic layer in ALD and an organic layer from CVD. The need to move the samples in and out of the chambers may compromise the film quality, but also limit the manufacturability of potential configurations consisting of many alternating layers. An emerging technology, molecular layer deposition (MLD), may be one of the candidates to address these issues. MLD enables deposition of organic components in the high-quality thin films with molecular precision on the thickness and composition, with potential combination with ALD to create multi-layered organic/inorganic high-quality coatings in the same chamber [165].

This multi-combinational pathway could be pursued more effectively once the exact failure mechanisms of each material and the properties of the inter-material interfaces are well established. Optimization of the deposition process and film thickness would be another future challenge. For all the materials in this review, some degree of performance variation is observed even with the same physical characteristics of the encapsulations. This is attributed to the difference in the film quality of the coatings which greatly depends on the deposition parameters, such as pressure, temperature, deposition rate, surface topography of the test device and its pre-deposition treatment, even with the same film thickness from the same equipment. Standardization of the deposition process is thus a critical requirement for regulatory approval and clinical application of these emerging materials.

Dependence on the reliability on the coating thickness is another issue. It was suggested that an Al_2_O_3_ coating thicker than 32 nm did not make a noticeable difference in barrier performance [30]. For LCP, the film thickness was determined by the available commercial products in the range of 25–100 μm in thickness, but the film thickness did not significantly affect the barrier property because the water penetration via the LCP-LCP bonding and LCP-metal bonding is the dominant mechanism rather than bulk penetration through the film surface. The HfO_2_ coating was not dissolved in accelerated aging test even after the failure of the device, suggesting that the water leakage occurred through nanoscale pinholes or defects rather than the bulk dissolution. In these cases, the MTTF of the encapsulation is less likely scaled up with the thickness of materials. On the other hand, for SiO_2_ and SiC, which actually dissolves away in a saline environment with a certain dissolution rate, the MTTF will be dependent on the encapsulation thickness, which can be optimized or compromised for targeting lifetime of the coating. Nevertheless, more systematic studies need to be conducted to optimize the coating thickness.

Although not included in this paper, it is also noteworthy to mention other emerging approaches including polyisobutylene (PIB) and miniaturized titanium packaging. PIB is a gas impermeable rubber which is used as pharmaceutical stoppers and blood bags for its inertness and excellent barrier properties. Recently, several works have demonstrated PIB for implantable devices such as intracranial pressure sensors, stents, and optogenetic stimulators [166,167,168], but its long-term reliability needs to be investigated for chronic implantation. While miniaturization of traditional titanium package for further reduced form factor and higher feedthrough density is also being pursued by means of advancement in precision machining and brazing, its compatibility with microfabricated devices is yet to be solved [169,170].

Shape memory polymer (SMP) is another interesting subject in the field of neural interfaces. Metastable shape is stored in SMP which can return to the globally stable shape by environmental triggers such as temperature, humidity or light [171]. These mechanically dynamic properties have been utilized for three-dimensional electrode arrays including folding, rolling, or extending structures [172,173,174], self-softening electrodes [173], or for surgical sutures [175]. There remain challenges in their long-term validation of biocompatibility and stability in body fluids.

## Figures and Tables

**Table 1:** Summary of selected results from long-term reliability tests of encapsulation with various inorganic materials.

Material ^1^	Deposition Method	Thickness	Testing Temp. (°C)	Test Samples	Measured ^2^	Failure Criteria	Results	^1^ Lifetime at 37 °C ^3^ (Years)	Ref.	Remarks
Al_2_O_3_ (+ ^1^PA)	ALD + CVD	52 nm + 6 μm	80	IDE	LC	>1 nA	>180 days *	>9.71	[30,32]	* Did not fail for 185 days
	ALD + CVD	52 nm + 6 μm	57	Wireless UEA interface	RF signal strength	Signal loss	* >465 days	5.10	[28]	* Without powering, did not fail for 465 days
							** 35 days	0.38		** continuous 5 V DC powering ×10 longer than PA-only
	ALD + CVD	52 nm + 6 μm	60	IDE	LC	>1 nA	214.6 days	2.90 *	[23]	* Averaged MTTF of XX samples
	ALD + CVD	52 nm + 6 μm	67	t-type IDE	LC	>1 nA	450 days	9.86	[26]	
				UEA			510 days	11.18		
PI/HfO_2_/Al_2_O/HfO_2_/PI	ALD, spin (PI)	16 μm/8 nm/20 nm/8 nm/16 μm		Films	WVTR	-	* <0.5 mg/m^2^ day	-	[33,34]	* Below detection limit, Cf. 4300 for PI-only, 4 for PI/Al_2_O_3_/PI
HfO_2_	ALD	100 nm	87	IDE	LC	>1 nA	126 days	11.1	[29]	* Five repetitions of (10 nm HfO_2_ + 10 nmSiO_2_)
HfO_2_/SiO_2_	ALD	* 100 nm		IDE			170 days	14.9		
HfO_2_/SiO_2_	ALD	* 100 nm		RF chips	Backscattered signal	Signal loss	185 days	16.2		
SiO_2_	Thermal	100 nm	90	Film	DR	-	80 nm/day	* 70	[35]	* Estimated from results of four temperatures
		1 μm	96	Electric components	Functionality	-	12 days	* 70		
	Thermal	511 nm	87	Photodiode	DR	-	0.104 nm/day	-	[36]	
	LPCVD	520 nm	90	Films	DR	-	1 nm/h		[37]	No dissolution at 37 °C for 22 weeks
SiC	PECVD	694 nm	87	Photodiode	DR	-	-	-	[36]	No dissolution for 16 weeks
	PECVD	67 nm	90	Films	DR	-	0.1 nm/h		[37]	No dissolution at 37 °C for 40 weeks
	PECVD	650 nm	90	IDE	EIS	-	-	-	[38]	No dissolution/defect for >6 weeks

^1^ PA: Parylene C; PI: polyimide; ^2^ LC: leakage current; DR: dissolution rate; WVTR: water vapor transmission rate; EIS: electrochemical impedance spectroscopy; ^3^ Estimated using the 10-degree rule from results and test conditions presented in the references unless otherwise stated.

**Table 2:** Properties of organic encapsulation materials for implantable devices.

Properties of Materials	Polyimide ^1^	Parylene C ^2^	Silicone Elastomer ^3^	LCP ^4^	SU-8 ^5^	COC ^6^
Encapsulation method	Spin coating	CVD	Casting, spin coating	Thermal bond	Spin coating	Thermal bond
Tensile strength (MPa)	128	69	6.7	180–190	73	46
Elongation (%)	10	200	305	30–40	4.8	1.7
Thermal expansion coefficient (ppm/°C)	40	35	340	15–18	52	60
Density(g/cm^3^)	–	1.289	–	–	1.075–1.153	1.02
Moisture absorption (%)	2-3	–	–	0.04	0.55	<0.01
Melting temperature (°C)	–	290	–	280–310	-	240–300
Glass transition temperature (°C)	>320	–	–	82–280	200	–
Dielectric coefficient	3.3 (1 kHz)	3.1 (1 kHz)	2.68 (100 kHz)	3.3 (2.8 GHz)	3.28 (1 GHz)	2.35 (1–10 kHz)
Resistivity (Ω∙cm)	10^16^	8.8 × 10^16^	2.9 × 10^14^	2 × 10^16^–3 × 10^16^	7.8 × 10^14^	>10^14^
Refractive index	1.7	1.639	1.3997–1.4225	–	–	1.53
UPS class	-	IV	VI	VI	-	VI

^1^ HD MicroSystems, PI-2525 [79], ^2^ Specialty Coating Systems, Parylene C [80], ^3^ Dow Corning, Sylgard 184 [81], ^4^ Kuraray, Vecstar [82], ^5^ Microchem, SU-8 3000 [83], ^6^ TOPAS, COC 5013 [84].

**Table 3:** Summary of selected results from long-term reliability tests of various organic encapsulation materials.

Material	Encapsulation Method	Thickness	Testing Temp. (°C)	Test Samples	^1^ Measured	Failure Criteria	Results	^2^ Lifetime at 37 °C (Years)	Ref.	Remarks
Polyimide	Spin	10 μm	75	IDE	LC	>1 μA	66 days	2.52	[85]	
	Spin	6.4–7.6 μm	37, 60	Film	* Mechanical properties		** No change	-	[86]	* e.g., Young’s modulus, stress and stress at break, fracture energy ** After 20 months of soaking
			85				** Degraded		[86]	
	Spin	10 μm	60	Electrode array	EIS	Loss of working channel	* 400 days	* 5	[87,88]	* Best two cases out of six samples
Parylene C	CVD	10 μm	75	IDE	LC	>1 μA	117 days	4.46	[85]	
	CVD	40 μm	85	Electrode array	Inter-line Resistance	50% change	31 days	2.4	[89]	
			97				15 days	2.7		
	CVD	6 μm	60	IDE	LC, EIS	>1 nA	49.1 days	0.66	[23]	
	CVD	6 μm	57	IDE	LC	>1 nA	150 days	1.64	[30]	
	CVD	6 μm	67	t-type IDE	LC	>1 nA	110 days	2.4	[26]	
Silicone elastomer (+Parylene C)	Dip (+CVD)	5 mm + 40 μm	85	Electrode array	Impedance	50% change	82 days	6.3	[89]	
			97				40 days	7.0		
PDMS	Spin	150 μm	36.5	Electrode array	EIS	-	* 54%	-	[90]	* Working electrode ratio relative to initial values after 209 days of soaking
Parylene-caulked PDMS	Spin + CVD						* 78%	-		
LCP	Thermal bonding	25 μm	75	IDE	LC	>1 μA	379 days	14.5	[85,91]	
		50 μm	87	Electrode array	* Waveform	>1 μA	114 days	10.0	[25]	
		25 μm		* IDE	LC		87 days	7.6		IDE in a mock package
		25 μm	75	IDE	LC	>1 μA	185 days	7.1	[92]	
				* IDE + MI			224 days	8.6		* MI: mechanical interlocking structure

^1^ LC: leakage current; DR: dissolution rate; ^2^ Estimated using the 10-degree rule from results and test conditions presented in the references unless otherwise stated.
